# Zoborg: On‐Demand Climbing Control for Cyborg Beetles

**DOI:** 10.1002/advs.202502095

**Published:** 2025-06-05

**Authors:** Lachlan Fitzgerald, H. Nhan Le, Robbie S. Wilson, H. Duoc Nguyen, Thanh Nho Do, T. Thang Vo‐Doan

**Affiliations:** ^1^ School of Mechanical and Mining Engineering The University of Queensland Brisbane QLD 4072 Australia; ^2^ School of the Environment The University of Queensland Brisbane QLD 4072 Australia; ^3^ School of Mechanical and Aerospace Engineering Nanyang Technological University Singapore Singapore 639798; ^4^ Graduate School of Biomedical Engineering Faculty of Engineering and Tyree Institute of Health Engineering (IHealthE) UNSW Sydney Sydney NSW 2052 Australia

**Keywords:** biohybrid robots, climbing, cyborg insects, electrical stimulation, insect‐scale robots, zophobas morio

## Abstract

Transitioning from horizontal surfaces to vertical walls is crucial for terrestrial robots to navigate complex environments. Replicating such impressive surface transitions in artificial insect‐scale robots has been particularly challenging. Here, innovative control schemes are introduced that enable ZoBorg (a cyborg beetle from *Zophobas morio*) to successfully climb walls from horizontal planes. The flex‐rigid structure, flexible footpads, sharp claws, and embedded sensors of the living insect enable ZoBorg to achieve agile locomotion with exceptional adaptability, all at low power and low cost. ZoBorg crosses low‐profile obstacles (5 and 8 mm steps) with a success rate exceeding 92% in less than one second. Most importantly, electrical stimulation of the elytron enables Zoborg to transition onto vertical walls with a success rate of 71.2% within 5 s. ZoBorg has potential applications for search and rescue missions due to its ability to traverse complex environments by crossing various obstacles, including low‐profile steps, inclines, and vertical walls.

## Graphical abstract

A novel on‐demand climbing protocol enables Zoborg (a cyborg beetle from the living *Zophobas morio*) to seamlessly transition from horizontal floors to vertical walls. Multiple unrestrained mechanosensors allow wall sensing while electrical stimulation of the elytron initiates climbing. Leveraging the agile locomotion and exceptional adaptability of the living insect, this approach ensures efficient navigation in complex environments.

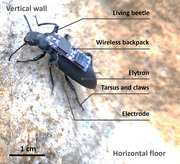

## 1 Introduction

Insect‐scale robots, due to their small size, are considered potential candidates that could one day penetrate rubble of collapsed structures to search for survivors in post‐disaster scenarios.^[^ ^1^, ^2^ ^]^ Post‐disaster environments are often complex, including both steep inclines and walls, which require robots to climb over obstacles and transition from one plane to another efficiently. Cyborg insects, or insect‐machine hybrid robots, offer an alternative to artificial insect‐scale climbing systems by using living insects as robot platforms, bypassing some of the challenges in actuation, sensing, and control of artificial robots.^[^ ^3^, ^4^ ^]^ Insects possess natural actuators (muscles), sensors, and control systems (brain and central nervous system) that enable complex, adaptive locomotion through comprehensive environmental interactions, making them masters of climbing. Insects also have natural adhesion mechanisms, including claws and adhesive pads, which enable secure gripping on diverse surfaces.^[^ ^5^, ^6^ ^]^ Their ability to adaptively adjust their centre of mass during coordinated body and leg movements ensures smooth and stable transitions without the need for complex external control systems.^[^ ^7^, ^8^, ^9^, ^10^ ^]^ The integration of programmable controls through cyborg systems further enhances these natural capabilities, enabling precise directional guidance and task‐specific behaviors.

Although there is significant progress in locomotion and navigation control for various insect platforms, climbing control remains to be demonstrated. Notable advances in locomotion control show electrical stimulation of leg muscles could elicit different walking gaits in living beetles,^[^ ^11^ ^]^ while that of antennae could induce turning, deceleration, and backward walking in cockroaches and darkling beetles.^[^ ^12^, ^13^ ^]^ Forward walking and acceleration have been achieved by stimulating both elytra of darkling beetles^[^ ^14^, ^15^ ^]^ or cerci of cockroaches.^[^ ^16^, ^17^ ^]^ Stimulating the individual elytron of darkling beetles could also induce sideways walking^[^ ^14^, ^15^ ^]^ while stimulating hind leg muscles and cerci in locusts could trigger jumping.^[^ ^18^, ^19^ ^]^ Both manual and feedback control systems can navigate cyborg insects toward defined destinations or along defined paths.^[^ ^20^, ^21^, ^22^, ^23^, ^24^, ^25^, ^26^ ^]^ To date, locomotion and navigation control of cyborg insects has primarily been limited to horizontal surfaces. Recent cyborg insect applications have shown the ability to climb slopes and navigate low‐profile obstacles within antennal reach. However, when encountering tall walls, these insects can only walk along them and avoid corners, lacking the capability to scale vertical surfaces.^[^ ^21^, ^27^ ^]^ Having the ability to transition surfaces while controlling a cyborg insect would vastly expand their reachable workspace. Combined with other advanced functions, such as on‐board human detection,^[^ ^21^ ^]^ steerable vision,^[^ ^28^ ^]^ and swarm technology,^[^ ^27^ ^]^ cyborg insects highlight their potential for future urban search and rescue applications.

Transitioning from horizontal to vertical surfaces poses complex challenges for both cyborg insects and artificial robots, including overcoming gravity, achieving stable adhesion, adapting to terrain changes, and coordinating sensor interactions.^[^ ^3^, ^29^, ^30^, ^31^ ^]^ Current limitations, such as restricted degrees of freedom, inadequate limb motion, and poor body articulation, hinder the performance of artificial robots.^[^ ^31^, ^32^, ^33^ ^]^ Effective adhesion strategies, including magnetism, suction, electro‐adhesion, dry adhesives, and sharp claws, have been explored, but they face energy efficiency challenges, especially during active gripping.^[^ ^3^, ^31^, ^34^, ^35^, ^36^ ^]^ Recent insect‐scale legged robots, such as RoBeetle^[^ ^32^ ^]^ and BHMbot,^[^ ^33^ ^]^ have demonstrated remarkable capabilities in locomotion and navigation, speeding up to 17.5 body lengths per second and successfully navigating complex environments. However, their climbing ability is limited to slopes of up to 15 ° due to the lack of appropriate attachment mechanisms on their legs. De Rivaz et al. ^[^ ^31^ ^]^ achieved impressive inverted and vertical climbing using the Harvard Ambulatory MicroRobot with Electroadhesion (HAMR‐E). This was possible through the integration of passive origami ankles, low‐voltage electroadhesive pads, and a parametric tripedal gait, allowing HAMR‐E to perform flexible locomotion on inclined and curved conductive surfaces, though it could not handle surface transitions. While strategies such as modifying adhesive pads, adding legs, and incorporating a compliant backbone or tail have been proposed to enhance the robot's ability to navigate complex 3D surfaces in the future, replicating the performance of real insects remains a significant challenge.^[^ ^31^, ^37^, ^38^ ^]^

Cyborg insects, with their natural agility, flexibility, and environmental adaptability, hold great potential to overcome these challenges.^[^ ^37^, ^39^ ^]^ However, achieving effective surface transitions in complex environments requires innovative strategies to integrate their natural capacities with artificial control, enabling seamless coordination and effective performance. Tran‐Ngoc et al.^[^ ^21^ ^]^ demonstrated that cyborg cockroaches with unrestrained antennae, controlled through cerci stimulation, can successfully traverse low‐profile obstacles shorter than the reach of their antennae to reach a destination within an enclosed arena. However, when tall walls were used, the cockroaches only followed the boundary of the arena without attempting to climb due to the lack of an effective climbing control protocol. Bai et al.^[^ ^27^ ^]^ demonstrated that swarms of cyborg cockroaches can navigate complex, obstructed environments, including low‐profile obstacles and slopes, but they were unable to scale vertical walls. Similarly, Ariyanto et al.^[^ ^20^ ^]^ proposed algorithms to help cyborg cockroaches avoid getting trapped in sharp corners by redirecting them; however, the algorithms did not address climbing, leaving the cockroaches vulnerable to entrapment in such scenarios. These recent advancements in navigation control for cyborg insects underscore both the challenges and the growing need for active wall‐climbing control. Guiding cyborg insects along or away from walls and corners may eventually lead them to their intended destination. However, this approach significantly increases travel time and risks trapping the insects within enclosed spaces, potentially preventing them from reaching their goal.^[^ ^20^, ^21^, ^27^ ^]^

**Figure 1:**
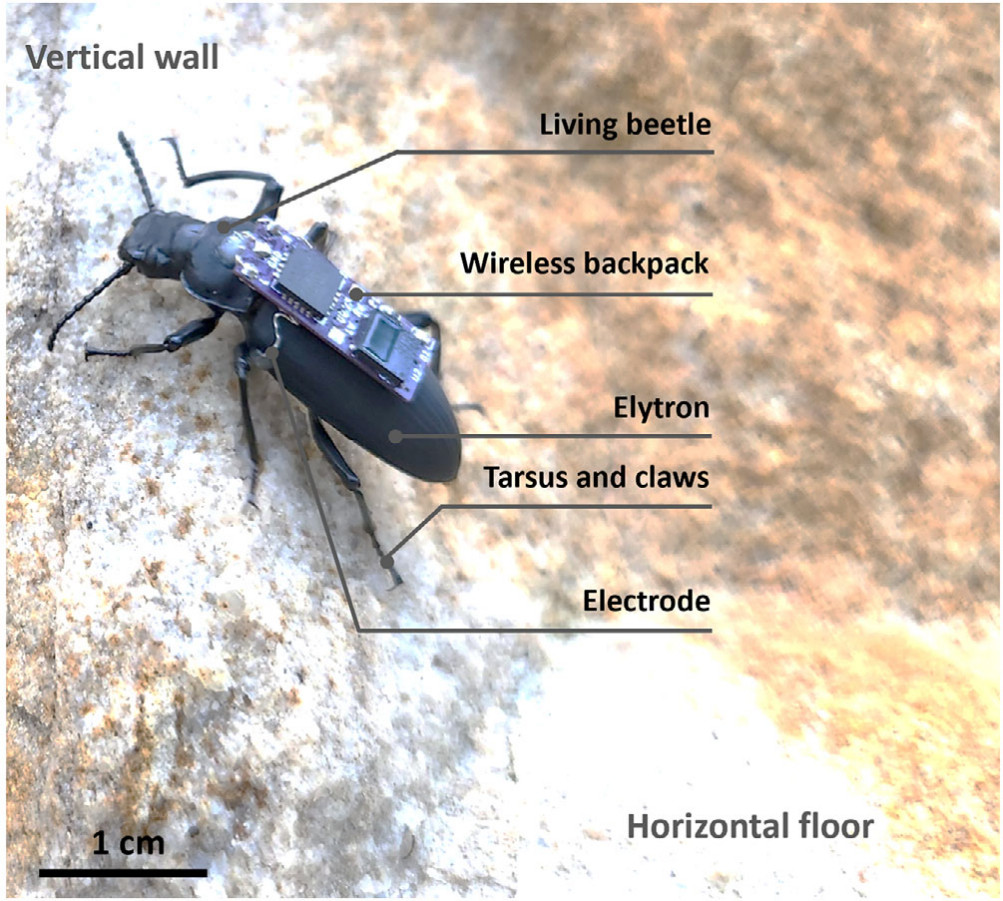
Overview of Zoborg. A wireless backpack was mounted on a living darkling beetle (*Zophobas morio*) with a working electrode implanted into each elytron and one counter electrode implanted into the pronotum of the beetle. Flexible tarsus and sharp claws enable Zoborg to climb over a rough surface like sandstone. Free antennae allow active interaction with the environment for more complex locomotion control.

The inability to transition from horizontal surfaces to vertical walls significantly limits the potential of cyborg insects in many applications. Herein, this paper demonstrates a novel on‐demand climbing protocol to successfully control the Zoborg (cyborg beetle from living *Zophobas morio*) to transit from horizontal floors to vertical walls (**Figure** **1**; Movie S1, Supporting Information). The proposed climbing protocol combines insect‐wall engagement and sideways motion of Zoborg elicited via elytron stimulation. When a Zoborg approaches an obstacle, the insect senses the obstacle's height through its mechanosensors and decides whether to cross, follow, or move away.^[^ ^8^ ^.^ ^9^ ^]^ Elytron stimulation is then triggered to push the Zoborg into the wall, forcing its contralateral side against the obstacle. The Zoborg could cross low‐profile steps (within the reach of its antennae) with ease, while transiting from horizontal floors to vertical walls required greater effort. The on‐demand climbing is achieved by leveraging the insect's natural tendency to move toward areas with less obstruction. Electrical stimulation of the elytron induces sideways motion toward the wall, where the vertical open space of the wall presents less obstruction, effectively promoting a transition from horizontal to vertical locomotion. On‐demand climbing capability is a critical advancement in enabling cyborg insects to navigate complex environments, which is necessary in search and rescue scenarios.

## 2 Results

### 2.1 Zoborg: a Cyborg Beetle from Living *Zophobas Morio*

Zoborg is a cyborg beetle developed based on living darkling beetles (*Zophobas morio*) (Figure 1). A wireless backpack consists of an ATTiny85V microcontroller, and an IR receiver was mounted on the insect to receive and send commands to the microcontroller to generate electrical stimulation (Figure 1; Figure S1, Supporting Information). One working electrode was implanted into the anterior edge of each elytron of the beetle, while the ground electrode was implanted into the pronotum from the dorsal side. The other ends of the electrodes were soldered to the stimulation and ground terminals of the backpack prior to the implantation. Once the backpack received the command from the IR controller, it generated an electrical pulse train to excite the sensory organs in the elytra and thus induced sideways walking of the Zoborg. The system has free antennae and other mechanosensors for assessing obstacles while its flexible tarsus with claws enables climbing on walls with rough surfaces like sandstone (Figure 1; Movie S1, Supporting Information).

### 2.2 Locomotion Control Via Electrical Stimulation of Elytra

**Figure 2:**
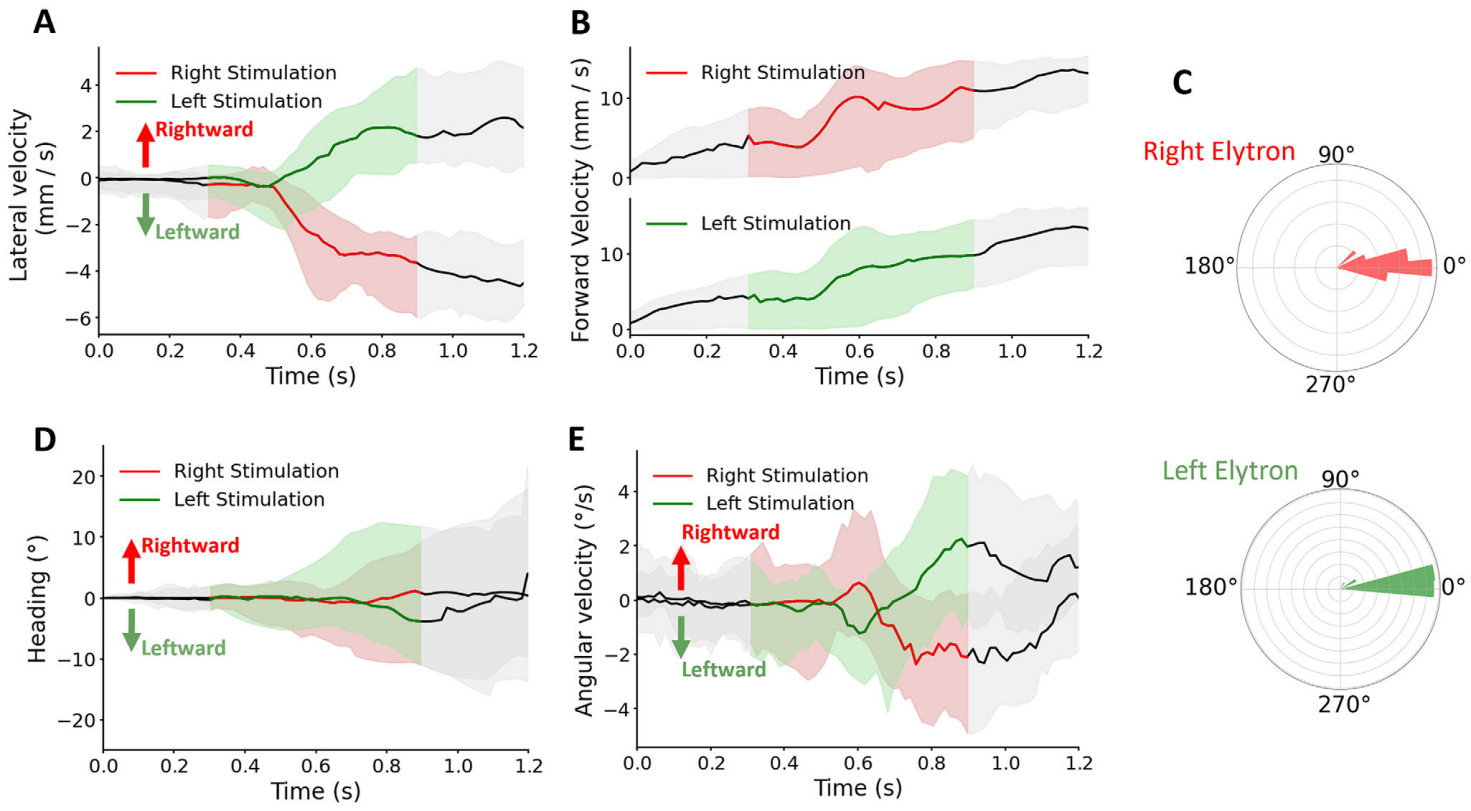
Response of Zoborg to electrical stimulation of the left and right elytron. A) Elytra stimulation induced sideways walking contralaterally to the side of stimulation. Right elytron stimulation elicits an increase in lateral velocity toward the left side, and vice versa. B) Stimulation of either left or right elytron induced an increase in the forward velocity of the insect. C) There are negligible changes in heading due to elytra stimulation as the induced angles are distributed ≈0. D) Changes in heading angle over time due to elytra stimulation. The electrical stimulation of left and right elytron shows negligible differences; beetles generally keep their heading angle consistent throughout stimulation. E) There are negligible changes in angular velocities when either the left or the right elytron was stimulated. Black lines indicate a non‐stimulation period, whereas the red and green lines indicate right and left elytron stimulation, respectively. Shaded regions indicate standard deviation (*n* = 3 beetles, *n* = 124 trials).

Electrical stimulation of the individual elytron drove the Zoborg contralaterally sideways while accelerating forward (Movie S2, Supporting Information; **Figure** **2**, N = 3 beetles, n = 161 trials). These responses resemble those previously observed for both tactile and electrical stimulation of the beetle's elytron, where the excitation of sensory neurons within the elytron triggered obstacle avoidance or escape behaviors, causing the beetles to move laterally.^[^ ^14^ ^]^ The beetle increased its sideways (lateral) velocity by an average of 2.84 mm s^−1^ (Figure 2A, *p* < 0.05, *t* = 7.73). The beetle also increased its forward velocity by 10.09 mm s^−1^ during the stimulation (Figure 2B, *p* < 0.05, *t* = −3.25). The change in heading angle of the beetles was insignificant (Figure 2C), as the beetles altered their heading ipsilaterally with an amount of only 2.30 ± 16.27 ° and −3.24 ± 16.94 ° at the end of the stimulation when left and right elytron were stimulated, respectively (Figure 2C,D). There were also minor ipsilateral increasements of below 3 degrees s^−1^ in angular velocities (Figure 2E). Thus, elytron stimulation induces contralateral sideways and forward velocity of the beetle, which is different from steering control by antennae stimulation, where the change in heading of the insects is dominant.^[^ ^12^, ^14^, ^15^ ^]^ Combining these controlled motions with the natural interaction between the antennae and environmental obstacles could enable new locomotion capabilities for the Zoborg, such as climbing, which relies on sensory input from the antennae to navigate and negotiate obstacles effectively.^[^ ^7^, ^40^, ^41^ ^]^

### 2.3 Crossing Low‐Profile Obstacles

**Figure 3:**
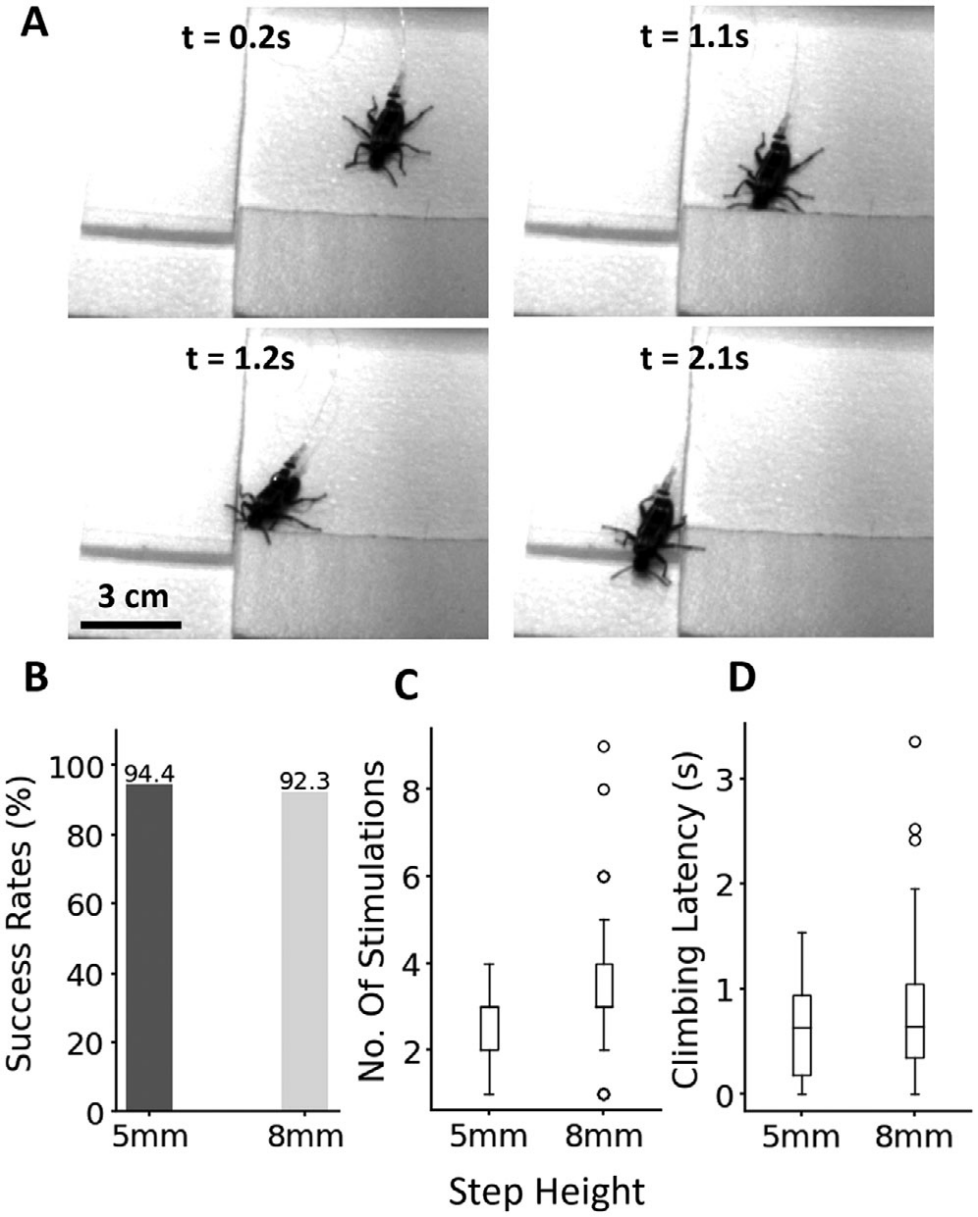
Performance of Zoborg when crossing low‐profile steps. A) Extracted frames when Zoborg was controlled to cross the 5 mm step (Movie S3, Supporting Information). The height of the beetle is ≈8 mm. Zoborg's various mechanosensors were able to reach the top of low‐profile obstacles, encouraging it to cross the steps when prompted by elytra stimulation. B) Success rates for crossing 5 mm (*n* = 5 beetles, *n* = 143 trials) and 8 mm steps (*n* = 5 beetles, *n* = 156 trials) were 94.4% and 92.3%, respectively. C) Number of stimulations required to successfully cross the 5 and 8 mm steps were 2.75 ± 0.92 and 3.46 ± 1.42, respectively (*p* = 0.006, *t* = −2.76). D) Latency for crossing 5 and 8 mm steps were 0.625 ± 0.48 and 0.75 ± 0.62 s, respectively (*p* = 0.275, *t* = −1.1).

Leveraging the innate obstacle negotiation ability of insects, Zoborg could cross low‐profile steps of 5 and 8 mm when elytron stimulation was used to drive the insect against those obstacles (**Figure** **3**; Movie S3, Supporting Information). Insects utilize various mechanosensors to explore obstacles, exhibiting a tendency to climb over barriers when their antennal reach exceeds the obstacle's height.^[^ ^9^ ^]^ Conversely, when obstacles surpass the antennal reach, insects are more likely to engage in wall‐following behavior.^[^ ^8^, ^40^, ^41^ ^]^ The 8 mm steps approximate the insect's height, while the 5 mm steps were notably shorter. In both cases, the beetle's antennae could extend beyond the height of the obstacles. Zoborg demonstrated remarkable agility in crossing low‐profile steps, achieving a 94.4% success rate for 5 mm obstacles (*n* = 5 beetles, *n* = 143 trials) and a 92.3% success rate for 8 mm obstacles (*n* = 5 beetles, *n* = 156 trials) (Figure 3B). This high success rate can be attributed to the insects’ ability to sense the steps’ height through their mechanosensors, thereby increasing the likelihood to continue in the desired direction and successfully navigate the obstacle. The minimal difference in success rates between the two step heights suggests that the beetles' climbing behavior remains consistent within this range of step sizes. The climbing performance was further evaluated through two criteria, control effort and climbing latency. Climbing latency was defined as the time taken for the beetle to entirely transition planes (all six legs on the obstacle) from the moment that the beetle engaged (made contact) with the obstacles. The control effort was measured as the number of stimulations that took place during this time. Zoborg exhibited a low control effort of 2.75 ± 0.92 and 3.46 ± 1.42 stimulations (Figure 3C, *p* < 0.05, *t* = −2.76) and a low climbing latency of 0.625 ± 0.48 and 0.75 ± 0.62 s (Figure 3D, *p* > 0.2, *t* = −1.1) when crossing 5 and 8 mm steps, respectively.

### 2.4 Novel On‐Demand Climbing Strategy: Transition from Horizontal Planes to Vertical Walls

In contrast to low obstacles, when encountering tall walls, information from multiple mechanosensors about the barrier's height can potentially signal that it is too challenging to overcome. Insects naturally tend to follow the path of least physical resistance,^[^ ^9^ ^]^ necessitating an external motivating force to induce wall‐climbing behavior. While antenna stimulation can effectively guide insects toward walls, it inhibits the engagement of their antennae with the environment.^[^ ^20^, ^21^ ^]^ Elytra stimulation offers a strategic advantage by preserving the insect's natural antennal function.^[^ ^14^, ^15^ ^]^ Combining wall engagement via the insects natural mechanosensors and sideways walking control via elytra stimulation, Zoborg would transition from horizontal floors to walls with open spaces, following paths of least resistance.

**Figure 4:**
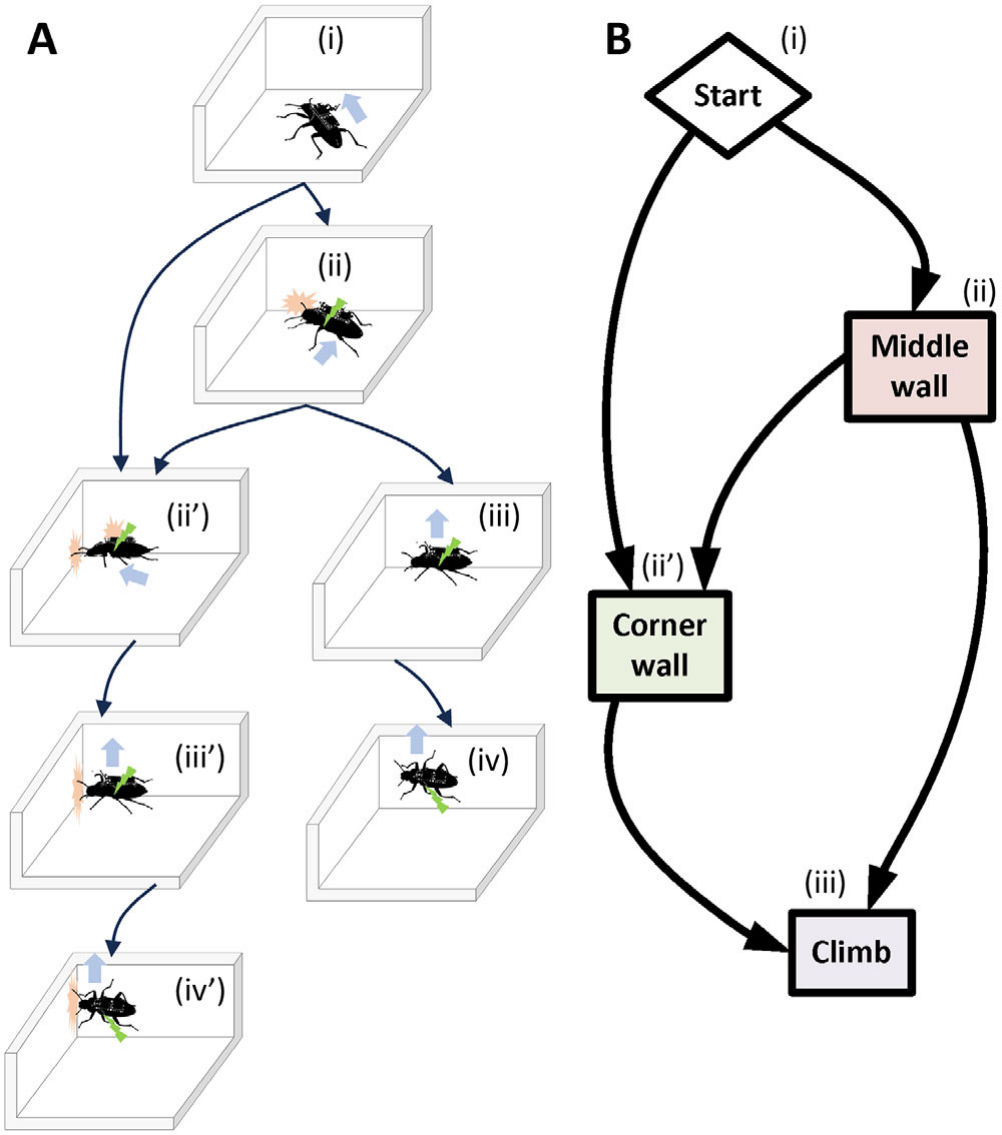
On‐demand climbing protocol. A) Illustrative cartoons demonstrating the novel on‐demand climbing protocol. i) Zoborg is controlled to engage with the wall. ii) Once the beetle engages with the wall, contralateral elytron stimulation (side opposite to that facing the wall) is triggered to align the insect parallel to the wall. iii) Contralateral elytron stimulation is maintained to push the Zoborg to transition into the wall. iv) Zoborg can then immediately climb or ii’) keep following the wall and climb upon reaching the corner. The first case is defined as middle wall climbing, where the induced sideways walking of the contralateral elytron stimulation provides sufficient impetus to elicit a change in planes at the point the beetle contacts the wall. The second case is defined as corner wall climbing, where Zoborg keeps walking forward, following the wall, until reaching the corner due to no obstruction in front of the beetle. iii’, iv') Upon reaching the corner, contralateral elytron stimulation pushes the Zoborg to transition into the wall due to the least restriction from the open area of the wall. Zoborg can identify the restriction from the wall and corners due to the engagement of multiple mechanosensors with the wall. B) A flow chart describing the two different paths for on‐demand wall climbing.

Thus, this paper proposes a novel on‐demand climbing protocol to transition the beetle from horizontal to vertical planes. The protocol integrates the engagement of the insect's mechanoreceptors, lateral walking control through elytra stimulation, and Zoborg's natural tendency to move toward areas of lower resistance (**Figure** **4**). This protocol consists of three phases:

#### 2.4.1 Approach and Engagement

The Zoborg is guided into the wall by increasing its lateral and forward velocity using elytra stimulation (Figure 4A‐i). Once the Zoborg reaches the wall, its mechanosensors allow it to characterize the wall precisely for planning subsequent motions. The climbing command is successful if the Zoborg transitions into the wall. Otherwise, the second phase is executed.

#### 2.4.2 Alignment

If the Zoborg does not climb upon contacting the wall, elytron stimulation on the outer side is triggered to align the insect's body parallel to the wall (Figure 4A‐ii). Aligning the insect's body parallel to the wall is critical for maximising the surface area in contact with the wall and the number of legs able to make initial contact. Aligning the insect parallel to the wall also maximises the effectiveness of the induced sideways movement, ensuring that this movement is directed entirely against the wall.

#### 2.4.3 Plane Transition

In the final phase, a series of contralateral elytron stimulations is applied to push the Zoborg to move sideways and transition into the wall, essentially the path of least resistance (Figure 4A‐iii,B‐ii). The climbing control is successful once all legs are positioned on the wall (Figure 4A‐iv,B‐iii). Elytra stimulation can also drive the Zoborg along the wall (Figure 4A‐ii’,B‐ii’), but eventually makes it transition onto the wall upon reaching a corner due to the increased spatial restrictions in the corner (Figure 4A‐iii’,iv’,B‐iii). Thus, the on‐demand climbing protocol enables the Zoborg to climb walls at either the midsection or the corners.

### 2.5 On‐Demand Wall Climbing Control

**Figure 5:**
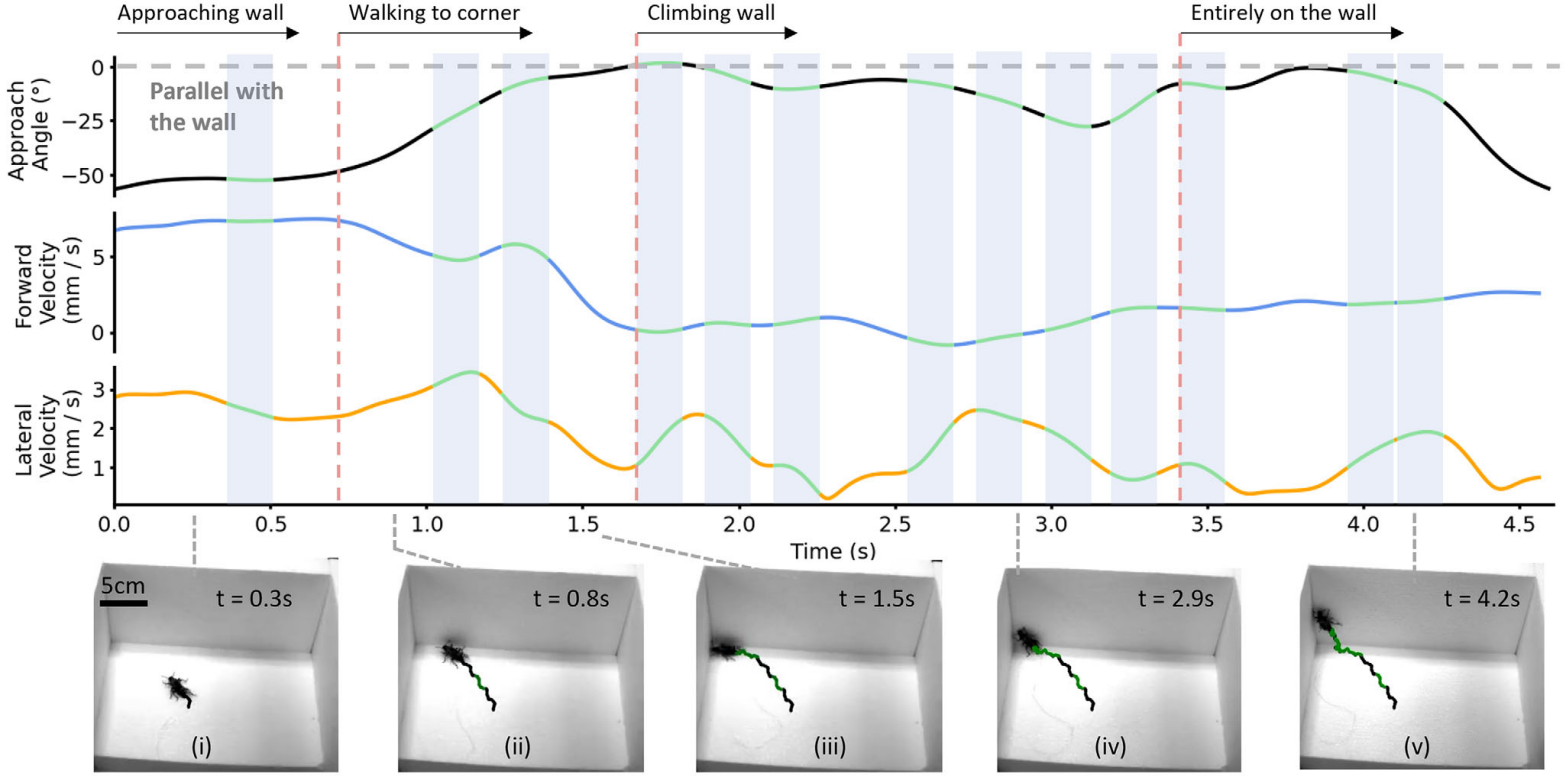
Representative trial of corner wall climbing. Changes in approach angle, lateral and forward velocity of the Zoborg during a corner wall climbing induced via left elytron stimulation. The bottom row presents extracted frames of beetle motion from Movie S4 (Supporting Information). Zoborg was controlled toward the wall. Upon reaching the wall (*t* = 0.8 s, image (ii)), left elytron stimulation was used to align the Zoborg parallel with the wall, which made the approach angle converge to 0 °. The forward velocity reduced to 0 mm s^−1^ once the Zoborg reached the corner (*t* = 1.5 s, image (iii)). Lateral velocity increased when the Zoborg was stimulated in open space and maintained low when the Zoborg followed the wall. The lateral velocity increased again when left elytron stimulation induced wall climbing in Zoborg. A successful climb was defined as all legs of the Zoborg positioned into the wall. Green lines and shaded regions indicate left elytron stimulation.

**Figure 6:**
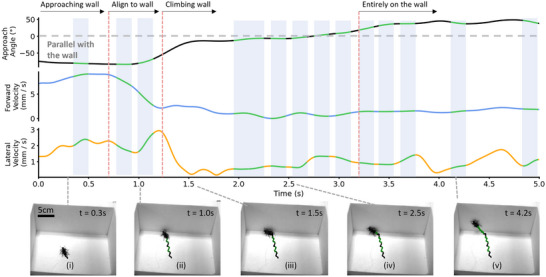
Representative trial of middle wall climbing. Changes in approach angle, lateral, and forward velocities of the Zoborg during a middle wall climbing trial included by left elytron stimulation. The bottom row presents extracted frames of beetle motion from Movie S4 (Supporting Information). Zoborg was controlled toward the wall using elytron stimulation. Upon reaching the wall (*t* = 1.0 s, image (ii)), left elytron stimulation was used to align the Zoborg parallel with the wall. Forward velocity reduced as Zoborg contacted the wall, as climbing behavior commenced at the point of contact with the wall (image (iii)). Lateral velocity increased when the Zoborg was stimulated in open space and when it was aligned with the vertical surface (*t* = 1.0 s), subsequently dropping after alignment with the vertical surface (*t* = 1.5 s). Lateral velocity began to increase again when Zoborg started climbing. A successful climb was defined as all legs of the Zoborg positioned into the wall. Green lines and shaded regions indicate left elytron stimulation.

Zoborg exhibited two distinct patterns of wall‐climbing behavior as proposed by the on‐demand climbing protocol, which were categorized as corner and middle wall climbing. Corner wall climbing was characterized by the Zoborg initially engaging with the vertical surface, subsequently navigating toward a corner of the arena, and then executing the plane transition within this corner (Figures 4A,B, **5**; Movie S4, Supporting Information). In middle wall climbing, Zoborg climbs the vertical wall at the point of initial engagement along the wall, excluding the corners (Figures 4A,B, **6**; Movie S4, Supporting Information). A representative corner climbing trial achieved through left elytron stimulation is illustrated in Figure 5 and Movie S4 (Supporting Information). Zoborg initiates contact with the vertical surface at *t* = 0.8 s (Figure 5 image (ii)), whereupon left elytron stimulation pushes the insect along the wall until it reaches a corner (Figure 5 image (iii)). At this juncture, Zoborg's approach angle converges to zero, and its forward velocity decreases. Subsequently, additional left stimulations induce climbing behavior (Figure 5 image (iv)) in Zoborg, facilitated by tactile information from mechanosensors impeding forward movement, while left elytron stimulation promotes lateral movement toward the wall, which has the lowest resistance. Lateral velocity can be seen to increase periodically during the climbing interval. Figure 6 and Movie S4 (Supporting Information) present a representative middle wall climbing trial also through left elytron stimulation. In this scenario, Zoborg establishes contact with the vertical wall at *t* = 1.0 s (Figure 6 image (ii)). Left elytron stimulation then aligns the insect body along the wall which make both forward velocity and approach angle immediately converge toward 0 while lateral velocity increases upon wall contact. Climbing behavior begins when the body aligns with the vertical surface, marking an approach angle, forward velocity, and lateral velocity close to zero. During the transition, lateral velocity increases up to 1.2 mm s^−1^, directed toward the wall, while forward velocity fluctuates ≈1 mm s^−1^ (Figure 6 image (iv)).

**Figure 7:**
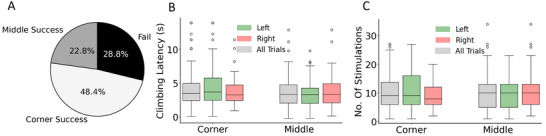
The performance of the on‐demand climbing protocol. A) Proportion of successful trials for corner and middle wall climbing (*n* = 6 beetles, *n* = 161 trials). There was a 71.2% success rate, where Zoborg performed an entire change of planes. B) The latencies, defined as the time from initial wall engagement to entire plane change (all 6 legs on vertical surface), were 4.3 ± 2.9 and 3.7 ± 2.35 s for corner and middle wall climbing, respectively. There is a negligible difference between corner and middle wall climbing latencies (*t*‐test: *p* > 0.2, *t* = 1.260). There is a negligible difference in climbing latencies when comparing stimulation side (*t*‐test: *p* > 0.45, *t* = 0.677). C) Control effort, the number of stimulations from initial wall engagement to entire plane change, were 10.6 ± 6.1 and 10.3 ± 6.2 for corner or middle wall climbing, respectively. There is a negligible difference between the control effort of corner and middle wall climbing (*t*‐test: *p* > 0.5, *t* = 0.400). There is a negligible difference in control effort when comparing left and right elytra stimulation (*t*‐test: *p* > 0.45, *t* = 0.718).

Wall climbing is more challenging than crossing low‐profile steps, evident in a lower success rate, higher latency, and higher control effort. Analysis of successful wall climbing attempts revealed that corner wall climbing occurred in 68% of trials, while middle wall climbing accounted for the remaining 32% (**Figure** **7**; Figure S2, Supporting Information, *n* = 6 beetles, *n* = 161 trials). Once the beetle engaged with the wall, the total success rate is 71.2%, with 48.4% from successful corner wall climbing and 22.8% from successful middle wall climbing (Figure 7A; Figure S3, Supporting Information). Although corner wall climbing might suggest low positional control in terms of wall‐climbing initiation, quantitative analysis of climbing latencies and control effort revealed negligible differences between the two modalities (Figure 7B,C; Figure S4A,B, Supporting Information, climbing latency: *p* > 0.2, *t* = 1.260, control effort: *p* > 0.6, *t* = 0.400). The latency for corner and middle wall climbing trials was 4.3 ± 2.9 and 3.7 ± 2.35 s, respectively (Figure 7B; Figure S4A, Supporting Information) while the control effort for corner and middle wall climbing was 10.6 ± 6.1 and 10.3 ± 6.2, respectively (Figure 7C; Figure S4B, Supporting Information). In addition, there are negligible statistical differences in climbing latencies and control efforts between left and right elytron stimulation (latency: *p* > 0.45, *t* = 0.677, effort: *p* > 0.45, *t* = 0.718). This similarity in latency and control effort across both climbing types suggests that the performance of the on‐demand climbing protocol remains consistent, regardless of the specific location on the wall where the transition occurs. Furthermore, this protocol demonstrated promising long‐term applicability, with the success rate remaining consistent at 76.3% five days after the implantation (Figure S3B,C, Supporting Information).

**Figure 8:**
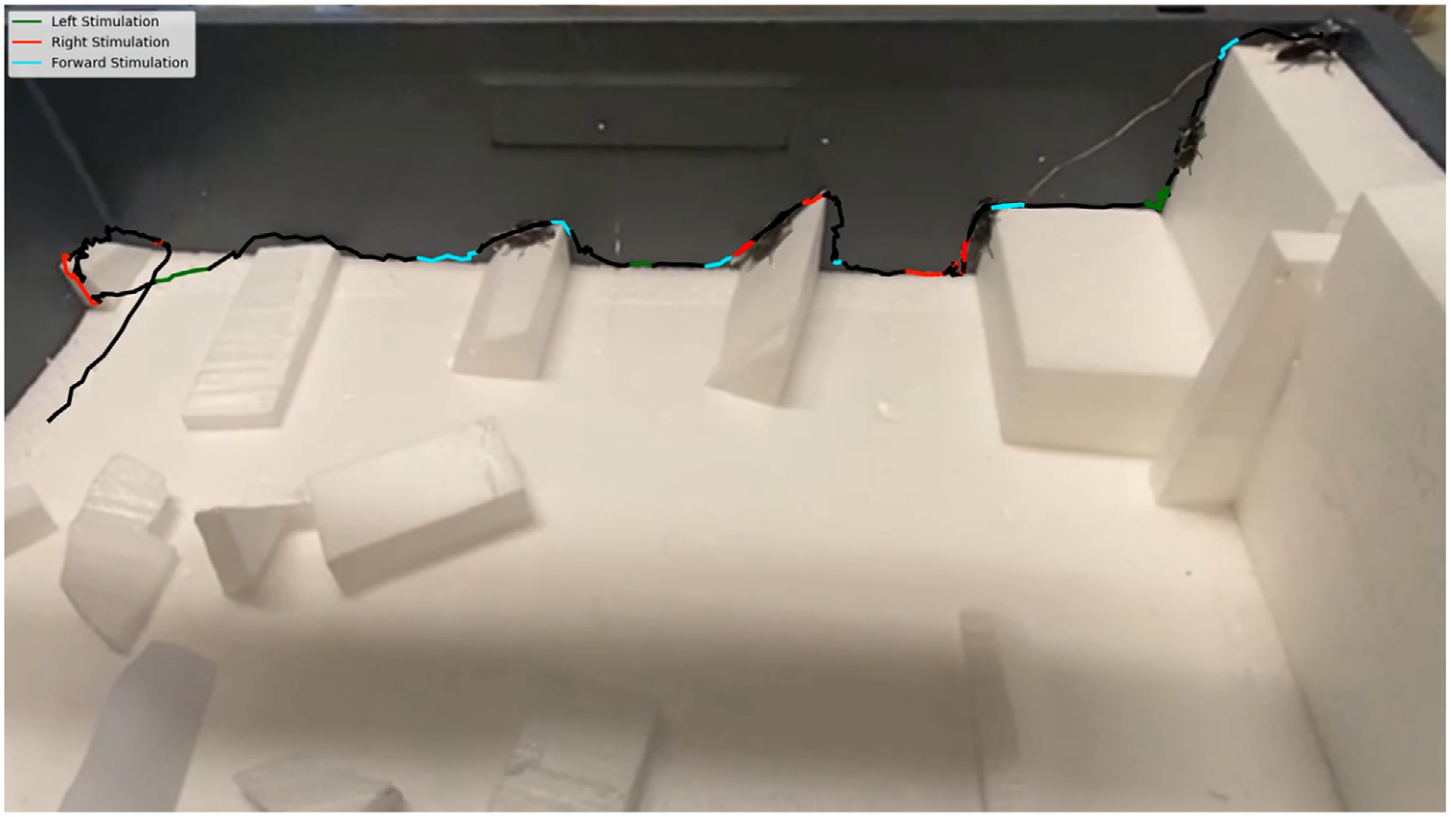
Navigation in a complex environment. The Zoborg was controlled to cross small obstacles, go up and down inclines, and climb vertical walls, demonstrating the diverse and practical use of elytra stimulation in controlling cyborg beetles over diverse and complex terrain. Green lines indicate stimulation of the left elytron, red lines indicate stimulation of the right elytron, and blue lines indicate periods of both elytra stimulation.

Zoborg also showed its capability to navigate complex environments (**Figure** **8**; Movie S5, Supporting Information). It crossed low‐profile obstacles and climbed over inclined surfaces with ease. Dealing with vertical walls, the Zoborg exhibited a significant increase in effort, but successfully climbed over. Zoborg was also demonstrated wirelessly with a battery for power supply. It was able to perform outdoor wall climbing on sandstone even when carrying a backpack (including battery) with a total weight around its own body weight (Movie S6, Supporting Information). Such demonstrations showcase the potential of Zoborg and cyborg insects in search and rescue operations, where agile locomotion, resilience, and adaptability are crucial for navigating complex environments.

## 3 Discussion

Cyborg insects, particularly ZoBorg, demonstrate significant advantages over current small‐scale walking robots in navigating complex terrains and transitioning from horizontal to vertical surfaces. While artificial insect‐scale robots have made strides in locomotion, including walking and climbing, the transition from horizontal surfaces to walls remains a formidable challenge.^[^ ^32^, ^36^, ^42^, ^43^ ^]^ This difficulty arises from the need for active foot pads, soft environmental interactions, and sophisticated sensing capabilities. In contrast, cyborg insects leverage millions of years of evolutionary adaptations, possessing natural actuators, sensors, and control systems that enable complex, adaptive locomotion through comprehensive environmental interactions.^[^ ^8^, ^23^, ^40^ ^]^ ZoBorg showcases impressive capabilities, with success rates exceeding 92% for crossing low‐profile obstacles (5 mm and 8 mm walls) typically within less than a second (Figure 3), and 71.2% for climbing walls within 5 s (Figure 7). This performance surpasses many current insect‐scale legged robots, which often struggle with rapid adaptation to varying terrain.

The preference for corner climbing over middle wall climbing (68% vs 32% of successful trials, Figure 7; Figure S2, Supporting Information) likely stems from a combination of factors. The electrical stimulation of the elytron produces not only a contralateral increase in sideways velocity but also an increase in the forward velocity of the beetle (Figure 2).^[^ ^14^, ^15^ ^]^ This increase in forward velocity may propel the insect along the wall, occasionally taking precedence over the increase in lateral velocity and guiding the insect toward a corner. Furthermore, insects often exhibit innate wall‐following behavior,^[^ ^41^ ^]^ which could explain why they continue along the vertical surface once aligned, until reaching a corner. Additionally, the fact that both antennae remain free during elytra stimulation allows the insect to utilize more of their innate sensing and control mechanisms. However, once the insect has aligned itself parallel to the vertical wall, its free antennae no longer detect obstacles ahead, making forward walking the path of least resistance. This combination of increased forward velocity, innate wall‐following behavior, and the lack of frontal obstacles detected by the antennae provides a compelling explanation for the preference for corner climbing over middle wall climbing.

Keeping the antennae free to sense is a crucial aspect of the on‐demand climbing protocol, despite potentially contributing to the preference for corner climbing over middle wall climbing. Insects rely on their antennae as vital sensory organs for initiating and guiding climbing behavior. Both stick insects and cockroaches have demonstrated that antennal sensing precedes climbing initiation, with obstacles being assessed by antennae and front legs before a decision to climb is made.^[^ ^7^, ^8^, ^40^ ^]^ However, the antennae are not the only mechanosensory organs that ZoBorg has at its disposal for object and environment classification. Insects are equipped with sensors on their legs and feet that can detect surface textures, vibration, and other physical stimuli.^[^ ^44^, ^45^ ^]^ Furthermore, insects have mechanosensors embedded within their exoskeletons that allow them to perceive forces and movements, enabling them to adapt their posture and locomotion in response to changes.^[^ ^46^, ^47^, ^48^ ^]^ For low‐profile obstacles, the beetles' antennae could detect the top of the steps in both cases. This sensory input, combined with the external motivation provided by elytra stimulation guiding the insect over the wall, resulted in the high success rates (> 92%) for obstacle crossing. Such proficiency in navigating low‐profile obstacles is particularly crucial for urban search and rescue scenarios, where debris and minor obstructions are common.

In the case of vertical walls, the mechanosensors allow the insect to detect obstacles higher than their body, enabling them to prepare for vertical climbing rather than crossing low‐profile obstacles. This sensory feedback allows the beetle to utilize its innate climbing ability, coordinating its legs as it would naturally. To improve positional control for wall climbing in Zoborg, antenna stimulation can be implemented by implanting electrodes at the base of the antennae, leaving the distal portions free for tactile exploration. This approach would enhance both directional control for locomotion and positional control for wall climbing, while preserving the insect's natural sensing capabilities. Antenna stimulation can emulate the presence of obstacles, creating a restraining effect in front of the insect, thereby promoting more efficient climbing along the middle of the wall. Furthermore, evaluating the effect of different stimulation parameters, such as amplitude, frequency, and duration, would give greater insight into the graded response and overall performance in climbing control.

The climbing performance of ZoBorg is, however, expected to be affected by surface roughness. Foam surfaces were used in the experiments to determine the effectiveness of the protocol by isolating other conditions like surface roughness, however climbing on natural substrates like sandstone (Movie S1 and Figure S6, Supporting Information) was also demonstrated and showed promising results. A systematic comparison across various surfaces is needed to fully understand the system's limitations and adaptability.

The current experimental setup for ZoBorg relied on a tethered power supply to prioritise the reliability and precision of the control mechanism and consistency during testing. However, we have demonstrated the feasibility of untethered climbing using a battery. ZoBorg was able to perform outdoor wall climbing with a battery payload of approximately its body weight (Movie S6, Supporting Information). This demonstrates the versatility of this system for untethered, practical applications and outlines its ability to carry significant payloads, which would be useful for the inclusion of other modalities, e.g. various sensing capabilities. Future studies would consider integrating compact and efficient onboard power systems to enhance mobility and versatility, addressing the challenges of payload capacity and prolonged operation in real‐world environments.

While the current demonstration of on‐demand climbing relied on manual control and video post‐processing to accurately reflect the performance of the protocols, future iterations of ZoBorg could further benefit from feedback control with onboard localization. By leveraging real‐time data from an Inertial Measurement Unit (IMU), a low‐cost and computationally efficient solution, the system could autonomously adjust climbing behaviors through closed‐loop control, correcting for disturbances such as slippage or misalignment with the wall. The IMU can detect when an insect engages in parallel wall‐following behavior by identifying consistent orientation and movement patterns indicative of proximity to a vertical surface.^[^ ^21^ ^]^ This information can be used to engage the on‐demand climbing protocol if necessary. Onboard cameras are another viable approach for characterizing the surrounding environment. Advances in lightweight and compact camera technology, including the development of wireless steerable vision for live insects and insect‐scale robots, make this feasible.^[^ ^26^, ^28^ ^]^ Combining visual data from an onboard camera with IMU‐based localization would create a robust multi‐sensor system capable of navigating diverse environments effectively. Incorporating feedback control based on these sensor inputs would enable ZoBorg to dynamically adapt its movement in response to environmental changes, significantly improving robustness and reliability during practical deployment.

In addition, this on‐demand climbing protocol provides significant insights for improving current artificial climbing robots, which often rely on inspiration from biological locomotion for enhancing their movement.^[^ ^49^, ^50^, ^51^ ^]^ When insects climb with their bodies perpendicular to the wall, they have to lift themselves to the greatest height, resulting in the highest potential energy according to the energy landscape framework proposed by Othayoth et al.^[^ ^52^ ^]^ In contrast, during sideways climbing, the insects maintain a posture closer to the ground, which likely involves lower potential energy. While this assumption about improved energy efficiency is consistent with the observed behavior, further experimental validation is necessary to confirm this hypothesis. Artificial robots could benefit from implementing similar strategies, using artificial feelers to detect surface orientation and adjusting their posture accordingly.

## 4 Conclusion

The on‐demand climbing protocol for Zoborg marks a breakthrough in the development of cyborg insects and insect‐scale robots, toward real‐life applications such as urban search and rescue. With a success rate of 71.2% across all climbing trials, this protocol demonstrates a remarkable achievement in the ability to control and navigate these biohybrid systems in complex environments. This success rate is particularly noteworthy given the challenges associated with transitioning from horizontal to vertical surfaces. Overcoming gravity, establishing stable adhesion, and rapidly adapting to abrupt changes in terrain orientation are challenging. The protocol's effectiveness lies in its ability to leverage the insects' natural biological advantages while enhancing them through precise artificial control via targeted stimulation. This synergistic approach allows the cyborg insects to transition from horizontal to vertical surfaces as well as crossing low‐profile obstacles effectively, combining their innate climbing abilities with artificial guidance for seamless performance.

## 5 Experimental Section

### Living Insect

Darkling beetles *(Zophobas morio)*, were used as platforms for developing ZoBorgs. The beetle has a rigid cuticle, small size (≈25–32 mm body length, and ≈8 mm height), light weight (≈750–950 mg), and long lifespan (≈3 months). This beetle is highly adaptable, that can cover a variety of terrains and has innate climbing abilities. The beetles were kept in large cages (Kmart modular storage drawer, 20.4 cm × 40 cm × 40 cm) with wheat bran bedding and were fed with fresh apple slices twice a week. After the experiments, the beetles were returned to the designated cages and continued to receive normal care for the rest of their life.

### Ethical Declaration

There is no requirement for ethics approval for experiments with insects. However, good practices were maintained when handling the insects to ensure their well‐being.

### Implantation

After being anesthetized on crushed ice, the beetle was delicately immobilized using clay under the microscope. An insect pin (#00, Indigo Instruments) was used to create a small hole in the middle of the pronotum and two holes in the anterior part of each elytron edge of the insect, ≈4 mm from the anterior edge (Figure S1A, Supporting Information). Three electrodes (127 µm diameter, bare; 178 µm diameter, Teflon‐coated silver‐wire; A‐M Systems) had their termini exposed by burning and subsequently implanted into the holes with a 2 mm depth (Figure S1A, Supporting Information). Melted beeswax was used to secure the electrodes after implantation. The three electrodes were soldered to the stimulation outputs and the counter output of the backpack, for elytra and pronotum implantations, respectively (Figure S1, Supporting Information).

### Wireless Backpack Stimulator

Zoborg uses a simple IR wireless backpack (15.8 mm × 5.8 mm, 160 mg) to receive commands and generate stimulation pulse trains. A microcontroller (ATtiny85V, Texas Instruments) was used to generate pulses and interface with the IR sensor. Pulse trains to the elytra were generated via two stimulation outputs, while command signals from the IR receiver (TSOP37238, Vishay) module were transferred via an input. The backpack receives an IR signal generated by an Arduino nano board connected to the computer. An LED was used to indicate the stimulation period (Figure S1, Supporting Information). The backpack was powered by a 3.3 V external power supply via a tethered connection or a 3.7 V Polymer Lithium battery (408 080, 25 mAh, 4 mm × 8 mm × 8 mm, 680 mg). The backpack was attached to the beetle using 3m double‐sided tape. The temperature of the backpack increased by only 1 °C during operation, which was unlikely to affect the natural behavior of the Zoborg, as *Zophobas morio* was an ectothermic species.^[^ ^53^ ^]^

### Electrical Stimulation

A square pulse train was employed to stimulate the insect's elytra. To clearly evaluate the efficacy of the on‐demand climbing protocol itself, stimulation parameters were fixed at 10 Hz frequency, 2 ms pulse width, and 600 ms duration. The stimulation voltage was maintained at 3.3 V. The Stimulation frequency was chosen to be 10 Hz as it closely matches the natural response to physical stimulation of *Zophobas morio*.^[^ ^14^ ^]^

### Experimental Rig and Motion Tracking

The experiment setup includes a climbing wall arena made of Styrofoam (280 mm × 250 mm × 150 mm), two cameras (Basler daA1920‐160 um, 8 mm lens) for video recording, two 850 nm LED rings for illumination, a Zoborg, an IR emitter controlled by an Arduino Nano board for sending commands to the Zoborg, and a PC for connecting and synchronizing those devices (Figure S5A,B, Supporting Information).

The Zoborg was controlled to climb walls or cross low‐profile steps manually while the videos and stimulation timestamps are recorded. Strand–Braid^[^ ^54^ ^]^ is used for recording and synchronizing videos from the cameras, while a custom Python software is used for interfacing with the user and sending the commands to Zoborg via the Arduino board. Videos of Zoborg's locomotion were tracked by DeepLabCut for the positions of head, pronotum, and tail.^[^ ^55^, ^56^ ^]^ 3D positions of those points were then reconstructed using Anipose.^[^ ^57^ ^]^ A custom Python software was then used for characterizing the climbing performance of the Zoborg (Figure S5C, Supporting Information).

### Data Analysis & Statistics

For evaluating the response of ZoBorg to elytra stimulation, 1.2 s of data points were extracted, including a 0.6 s stimulation period and 0.3 s before and after the stimulation had occurred. The data was then smoothed using an exponential weighted moving average filter with a smoothing factor of 0.25. The forward velocities (aligned with the body axis) and lateral velocities (perpendicular to the body axis) of the beetles were calculated by projecting the x and y components to those axes (Figure S6, Supporting Information). The positions of the pronotum and the tail of the insect were used for calculating the heading angle. The significance of the induced speed and change in heading angle was validated using a paired *t*‐test with a significance level of 0.05.

To evaluate the efficacy of the on‐demand climbing protocol, 3D positional data were gathered from the beginning of the trial to typically 1 to 2 s after the beetle had successfully transitioned planes. An exponential weighted moving average filter (smoothing factor 0.8) was applied to the extracted data before calculating velocities and approach angles of the beetle (Figure S7, Supporting Information).

Climbing latency was defined as the time taken for the beetle to entirely transition planes (all six legs on a vertical surface) from the moment that the beetle engaged (made contact) with the vertical surface. The control effort was measured as the number of stimulations that took place during this time. The statistical significance between climbing latencies and control efforts of different cases was validated using a paired *t*‐test with a significance level of 0.05.

## Conflict of Interest

The authors declare no conflict of interest.

## Author Contributions

L.F. and T.T.V.‐D. were responsible for conceptualization. L.F., H.N.L., and T.T.V.‐D. developed the methodology. Formal analysis was performed by L.F., H.N.L., H.D.N., and T.T.V.‐D. The investigation was conducted by L.F., H.N.L., and T.T.V.‐D. T.T.V.‐D. provided the resources. L.F. and T.T.V.‐D. handled visualization. T.N.D. and T.T.V.‐D. secured the funding. T.T.V.‐D. took care of project administration and supervision. The original draft was written by L.F., T.N.D., and T.T.V.‐D., while the review and editing were completed by L.F., H.D.N., R.W., T.N.D., and T.T.V.‐D.

## Supporting information



Supporting Information

Supplementary Movie S1

Supplementary Movie S2

Supplementary Movie S3

Supplementary Movie S4

Supplementary Movie S5

Supplementary Movie S6

## Data Availability

The data that support the findings of this study are available in the supplementary material of this article.
