# Supplementary material for: Zoborg: On‐Demand Climbing Control for Cyborg Beetles
Source: Adv Sci (Weinh). 2025 Jun 5;12(31):e02095. doi: 10.1002/advs.202502095 (PMC12376601; doi:10.1002/advs.202502095)
Supplement: Supplementary file 1 — Supporting Information [file ADVS-12-e02095-s002.docx]

Supporting Information

Zoborg: On-demand Climbing Control for Cyborg Beetles

Lachlan Fitzgerald, H. Nhan Le, Robbie S. Wilson, H. Duoc Nguyen, Thanh Nho Do, T. Thang Vo-Doan *

Supporting Methods

The effect of elytra stimulation was evaluated based on 2-dimensional data obtain from the top-view camera. The markers for pronotum and tail of the beetles were tracked and annotated as M and B, respectively (Figure S5). The heading angle $\theta$ and angular velocity $\dot{\theta}$ of the beetle were calculated as follows

$\theta= \frac{180}{\pi}\times\tan^{-1} \frac{M_{y}- B_{y}}{M_{x}-B_{x}}$ (1)

$\dot{\theta}= \frac{\Delta\theta}{dt}$ (2)

The forward velocity of the insect was calculated as the component of the velocity along the body axis, the line connecting points B and M (Figure S5). The velocity was scaled to mm/s based on insect’s body length (29.3 ± 3.5 mm). The forward velocity was calculated as:

$v_{f}=\frac{v\times cos\left( \theta- {tan}^{-1}\left( \frac{v_{y}}{v_{x}} \right) \right)}{dt}$ (3)

The lateral velocity ($v_{l}$) was perpendicular to the body axis.

$v_{l}=\frac{v\times sin\left( \theta- {tan}^{-1}\left( \frac{v_{y}}{v_{x}} \right) \right)}{dt}$ (4)

*dt* was taken as the time between frames, which was 1/100 s.

For evaluating wall climbing behaviour in 3-dimensions, the approach angle $\alpha$ (Figure S6) of the beetle was defined as the angle of the beetle with respect to the vertical wall, in the x-y plane. The angle $\alpha$ was adjusted to be relative to the vertical wall, as shown in Figure S6. The lateral and forward velocities were defined in the same manner as described above but adjusted for 3-dimensions (Figure S6), so that the velocities were representative of the beetle’s motion in 3-dimensions.

**Supporting Figures**

**
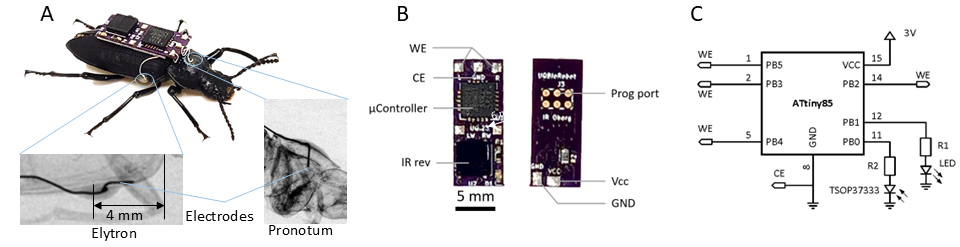
**

**Figure S1. Detailed assembly of Zoborg.** (**A**) ZoBorg with electrodes implanted in elytra and pronotum. X-ray images show depth and exact location of electrode implantation. (**B**) Top and bottom view of ZoBorg backpack. The backpack consists of a microcontroller (ATTiny85), an IR receiver, two working electrodes for elytra and a counter electrode for pronotum. The programming port is used for uploading the firmware to the microcontroller while Vcc and GND are used for tethered power or battery connection. (**C**) Schematic of the backpack.


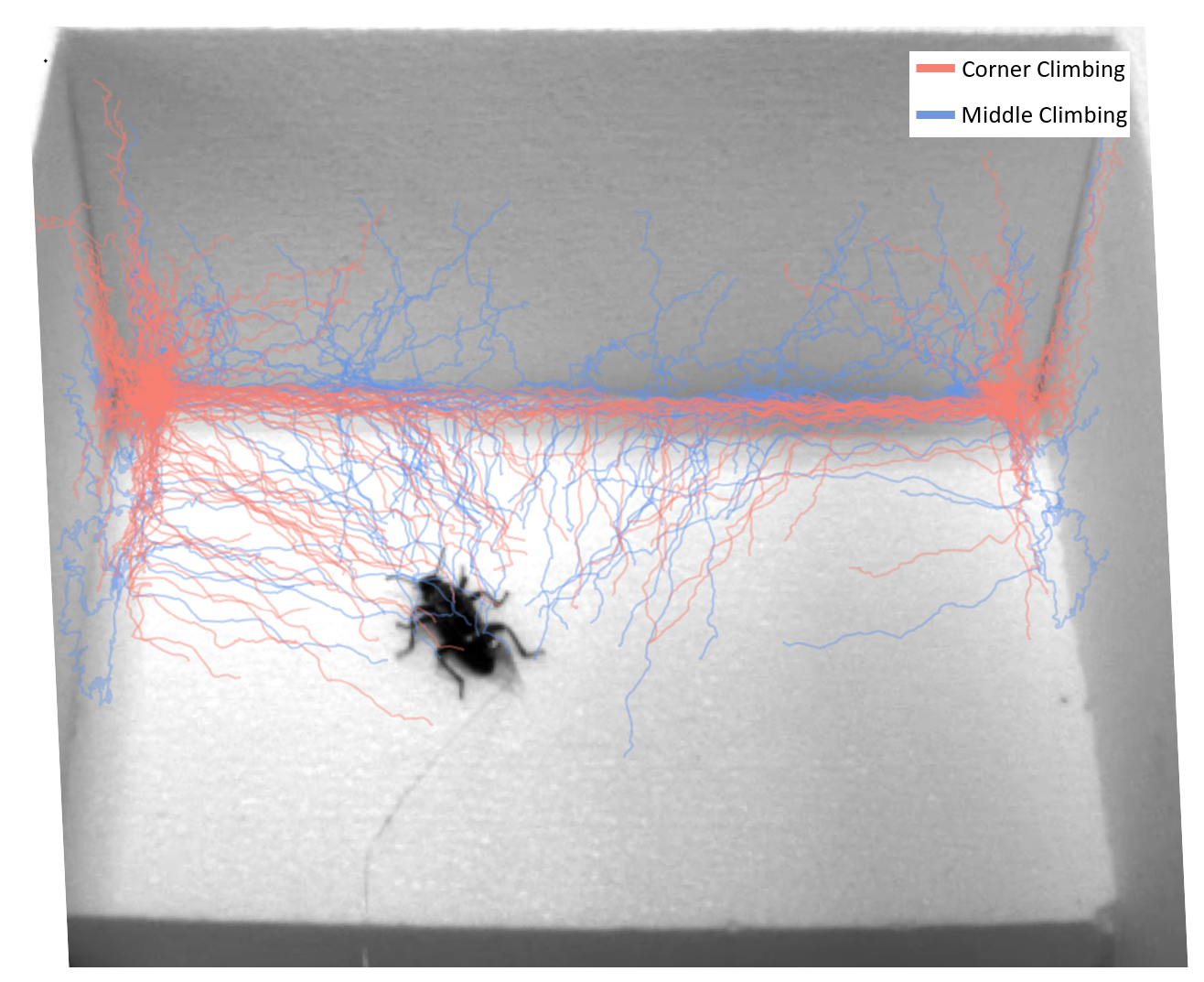
**Figure S2. Corner and middle wall climbing.** Representation of all successful on-demand climbing trials. Red lines indicate successful corner climbing trials and blue lines indicate successful middle wall climbing trials.


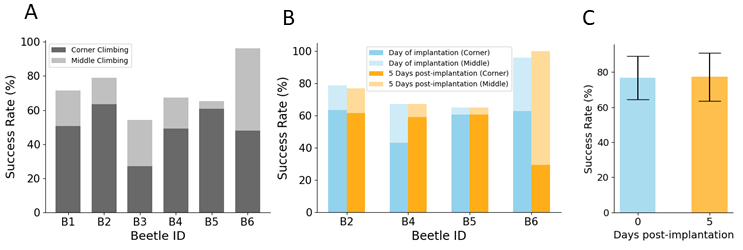


**Figure S3. The success rate of on-demand climbing across individual beetles and their long-term performance.** (A) Success rates of on-demand climbing across individual beetles, including the proportion of corner vs. middle wall climbing (N = 6 beetles, n = 161 trials). (B) Success rates of individual beetles performing on-demand climbing on the day of implantation and 5 days post-implantation, along with the corresponding proportions of corner and middle wall climbing (N = 4 beetles, n = 253 trials). (C) Overall success rates of the beetles on the day of implantation and 5 days after implantation.


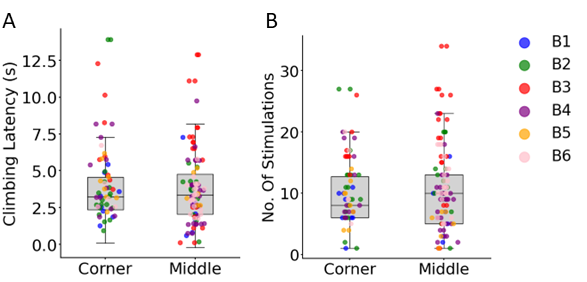


**Figure S4. Climbing latency and control effort for corner and middle wall climbing** (N = 6 beetles, n = 161 trials). (A) Overall climbing latencies of corner and middle wall climbing along with those from individual beetles. (B) Overall control effort of corner and middle wall climbing along with those from individual beetles. The box plots show the overall performance of the beetles while colour markers show data from individual trials of individual beetles.

**
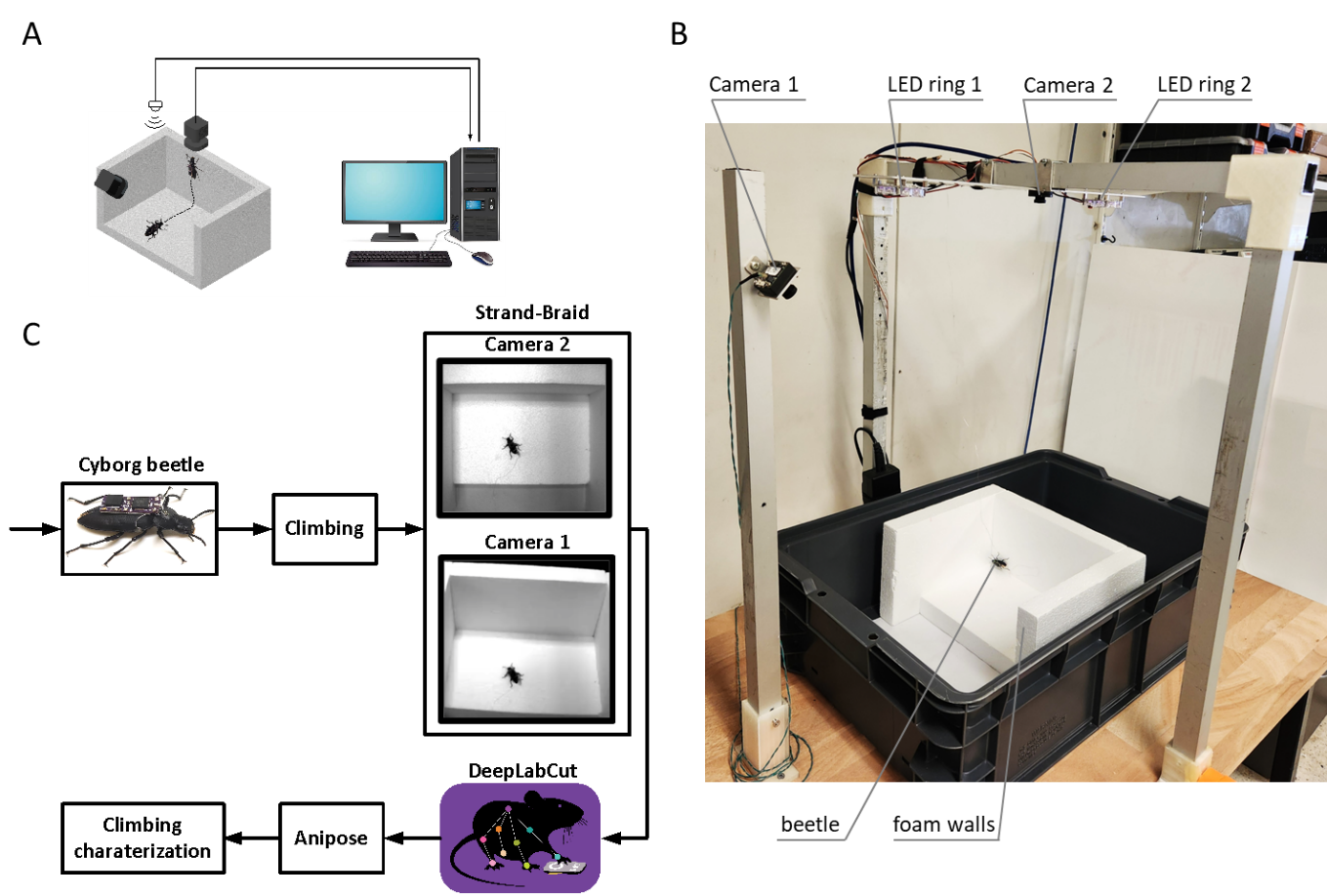
**

**Figure S5. Experimental Setup and Flow.** (**A**) Overview of the experimental setup. (**B**) The experimental rig (600 mm × 700 mm × 600 mm) includes an arena made of 3 vertical walls and a floor made of Styrofoam (280 mm × 250 mm × 150 mm). Two cameras were mounted on 25 mm aluminium profile for top-view and top side view of the arena. Two 850 nm LED rings were used for lighting. (**C**) Experimental flow for data collection and analysis. The cyborg beetle was controlled manually within the arena to perform wall climbing or step crossing. Videos of the two cameras were recorded and synchronized by Strand-Braid^[44]^ before tracking for positions of tail and pronotum of the beetle using DeepLabCut.^[45, 46]^ The 3D reconstruction of the climbing data was then triangulated using Anipose^[47]^ before being characterized for climbing performance by custom software.


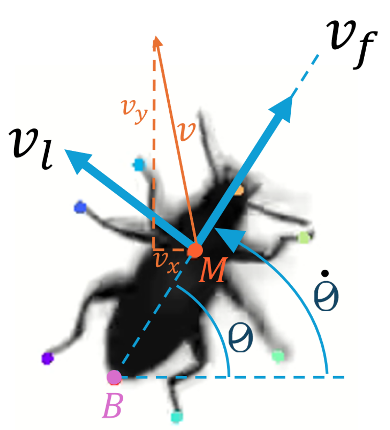


**Figure S6. Velocities and heading angle of the beetle.** Forward velocity (**v_f_**) and lateral velocity (**v_l_**) indicated along and perpendicular to the beetle axis aligned with BM, respectively. B is the marker for the tail while M is the marker for the pronotum. Forward and lateral velocities were calculated by projecting **v_x_** and **v_y_** to their axis. $\theta$ and $\dot{\theta}$ are heading angle and angular velocity of the beetle. All velocities were adjusted by constants to reflect speed in mm/s.


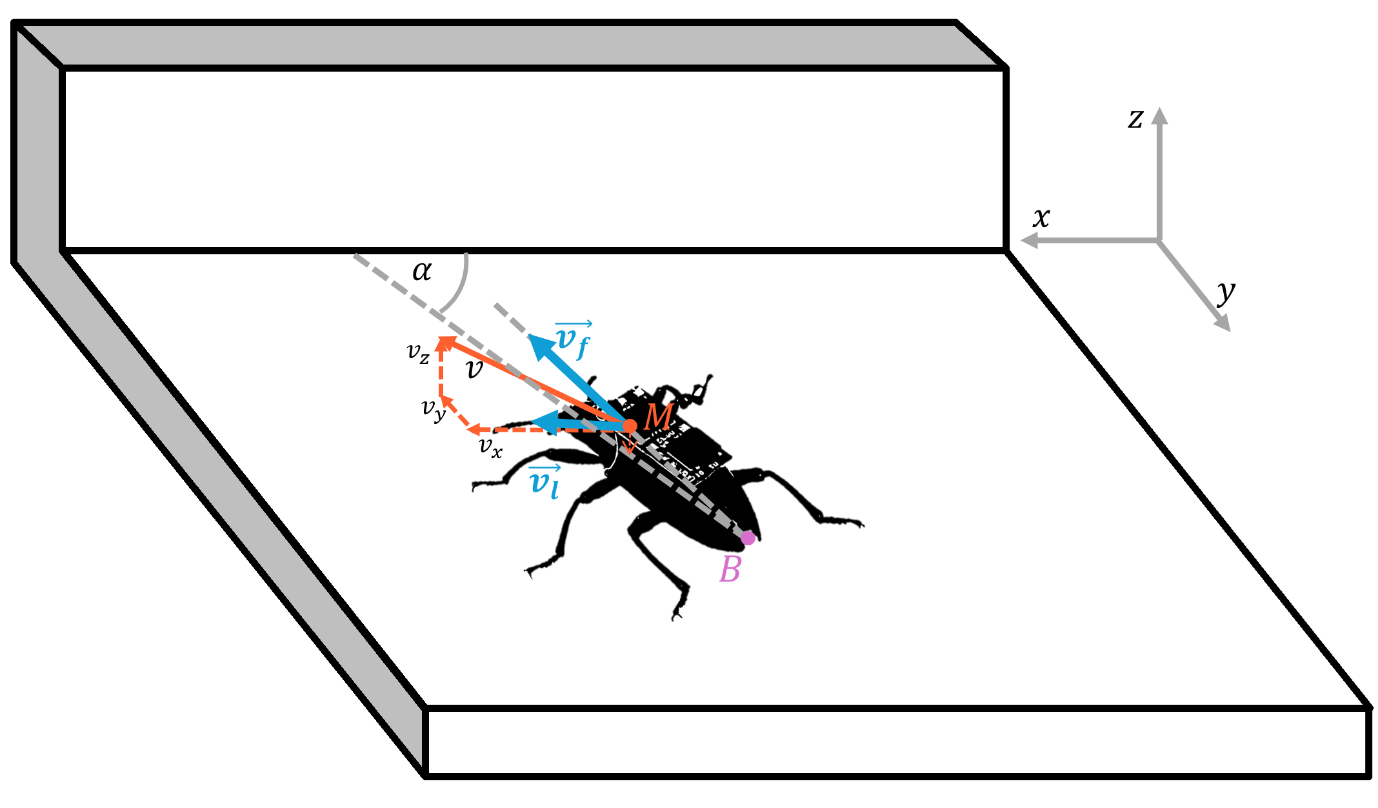
**Figure S7. Velocities and approach angle for climbing evaluation.** Forward and lateral velocities were calculated from the projections of **v_x_**, **v_y_**, and **v_z_**. The approach angle ($\alpha$) was defined based on a projection of the points B and M on the x-y plane only.

**Supporting Movies**

**Movie S1:** Demonstration of wall climbing on sandstone.

**Movie S2:** The effect of elytra stimulation on Zoborg’s locomotion. Electrical stimulation of individual elytron induced forward and contralateral sideways motion.

**Movie S3:** Representative demonstration of Zoborg crossing 5 mm and 8 mm steps.

**Movie S4:** Representative demonstrations of on-demand wall climbing control for Zoborg. The green lines indicate left elytron stimulation. Black lines represent periods without stimulation.

**Movie S5:** Demonstration of Zoborg navigating a complex environment. The green, red, blue, and black lines represent left elytron stimulation, right elytron stimulation, simultaneous elytra stimulation, and periods without stimulation, respectively. The movie is played at 3x speed.

**Movie S6:** Wireless demonstration of wall climbing on sandstone. The backpack is powered by a 3.7 V polymer lithium battery (408080, 25 mAh, dimensions: 4 mm × 8 mm × 8 mm, weight: 680 mg) securely attached to the beetle using 3M double-sided tape. The beetle weighs 920 mg, and the total weight of the backpack, including the battery, is 840 mg.
